# Visceral Leishmaniasis in a Resident of Swat, Khyber Pakhtunkhwa, Pakistan, Presenting to Civil Hospital Karachi: A Case Report

**DOI:** 10.7759/cureus.6059

**Published:** 2019-11-03

**Authors:** Shahzeen Saifullah Khan, Muhammad Hasan Shahab, Tahira Naz

**Affiliations:** 1 Pediatrics, Dow Medical College / Dr. Ruth KM Pfau Civil Hospital, Karachi, PAK; 2 Pediatrics, Dow University of Health Sciences / Dr. Ruth KM Pfau Civil Hospital, Karachi, PAK

**Keywords:** amastigotes, visceral leishmaniasis, kala-azar, amphotericin b, sodium stibogluconate, leishmania donovani, donovan bodies, leishmaniasis

## Abstract

Despite being prevalent in the cities of Gilgit-Baltistan and Azad Jammu Kashmir in north-eastern Pakistan, diagnosing visceral leishmaniasis for doctors in Pakistan can be an arduous task. We present here, a case of a five-year-old boy, who presented to us with a history of intermittent, high-grade fever, abdominal pain that was localized to the left side and abdominal distention as well as pallor for the past two and a half months. The child also developed measles in the week before presenting to us at Civil Hospital Karachi.

On examination, the child looked pale, with several hyperpigmented lesions on the face and nose. There was bilateral pedal edema, which extended upward to the thighs and generalized lymphadenopathy. The examination of the respiratory and cardiovascular system was normal. On examination of the abdomen, there was marked hepatosplenomegaly. A diagnosis of visceral leishmaniasis was made based on the findings of routine blood investigations indicative of pancytopenia, clinical manifestations, and epidemiology and, finally, a bone marrow biopsy report with demonstrable Donovan bodies. The patient's condition improved after five weeks of treatment with intravenous amphotericin B deoxycholate.

## Introduction

Visceral leishmaniasis is an oft-neglected and rampantly misdiagnosed, vector-borne parasitic disease caused by an obligate intracellular protozoan belonging to the genus Leishmania. It is transmitted by the bite of an infected female phlebotomus sand fly. The disease manifests in three forms: (1) visceral leishmaniasis (also known as Kala-azar), (2) cutaneous leishmaniasis, and (3) mucocutaneous leishmaniasis.

While cutaneous forms of Leishmaniasis afflicting patients belonging to the province of Khyber Pakhtunkhwa have been reported, there have been, to- date, no reported cases of visceral leishmaniasis from that province. The visceral form of leishmaniasis is, however, endemic in the north-eastern areas of Pakistan, especially Gilgit Baltistan and Azad Jammu Kashmir, with the most commonly reported causative species being Leishmania infantum [1].

The lifecycle of Leishmania begins with the infected sandfly injecting its promastigotes into the human host while taking a blood meal. Once in the bloodstream, the promastigotes are phagocytosed by macrophages, where they mature into amastigotes that continue to multiply within the cells belonging to the reticuloendothelial system and those of other tissues [2]. The phlebotomine vector of Leishmania is known to thrive in moist soils such as those of tropical rainforests and contaminated ones in animal shelters. They also seek shelter in overly crowded human habitations with poor sanitation [3].

Visceral leishmaniasis, if left untreated, can prove to be fatal. It clinically manifests as fever associated with hepatomegaly, splenomegaly, skin hyperpigmentation, pancytopenia, and weight loss. This understandably overlaps with other diagnoses like malaria, brucellosis, tropical splenomegaly syndrome, schistosomiasis, tuberculosis, and a myriad of other conditions with varying degrees of similar findings. As a result, any treatment directed at any other diagnosis will not yield clinical improvement. Therefore, the diagnosis, when suspected, is confirmed either by non-invasive serological tests, namely, direct agglutination tests (DAT) and lateral flow immunochromatographic tests (ICT), which are collectively referred to as rapid diagnostic tests (RDTs) or by the demonstration of the parasite in splenic or bone marrow aspirates [4]. Treatment is by the administration of intravenous amphotericin B, sodium stibogluconate (SSG), or miltefosine, depending on sensitivity.

## Case presentation

A five-year-old male child, weighing 13 kg, native to and born in the city of Swat, in the province of Khyber Pakhtunkhwa (formerly the North-West Frontier Province), Pakistan, was referred to us in June 2016 from the Children Cancer Hospital (CCH), complaining of prolonged fever, pallor, abdominal distention, and abdominal pain for the past two and a half months.

According to the child's uncle, the child was in his usual state of health two and a half months ago, when he developed fever, which was sudden in onset, high grade, documented as 102°F-104°F, associated with chills and rigors. There were no associated complaints of hematemesis, melena, diarrhea, vomiting, jaundice, dark-colored urine, worm infestation, petechiae, bruising, bone pain, or bleeding from any site. The absence of these complaints helped rule out other differential diagnoses in mind, such as malaria, enteric fever, dengue fever, schistosomiasis, leukemia, and lymphoma. The child, however, did complain of abdominal pain localized more towards the left hypochondrium. The complaints prompted the child's family to consult a local doctor in Swat but to no avail. The abdominal distention continued to worsen with time. The child also started to become paler day by day. This was associated with a decrease in appetite and significant weight loss. The family consulted another doctor in a local hospital, from where he was referred to CCH, Karachi.

While at CCH (now known as Indus Hospital-CCH), two to three packed red blood cells were transfused as the child was severely anemic. The child had already tested negative for tuberculosis, human immunodeficiency virus (HIV), and malaria. A* *bone marrow biopsy was carried out, which demonstrated histiocytes filled with organisms with a prominent nucleus and a rod-shaped para-nuclear kinetoplast, giving them a 'double-dot' appearance, characteristic of Leishmania Donovani bodies, confirming the diagnosis of visceral leishmaniasis. The patient was then referred for further treatment to Dr. Ruth KM Pfau Civil Hospital Karachi.

Note

**Figure 1:**
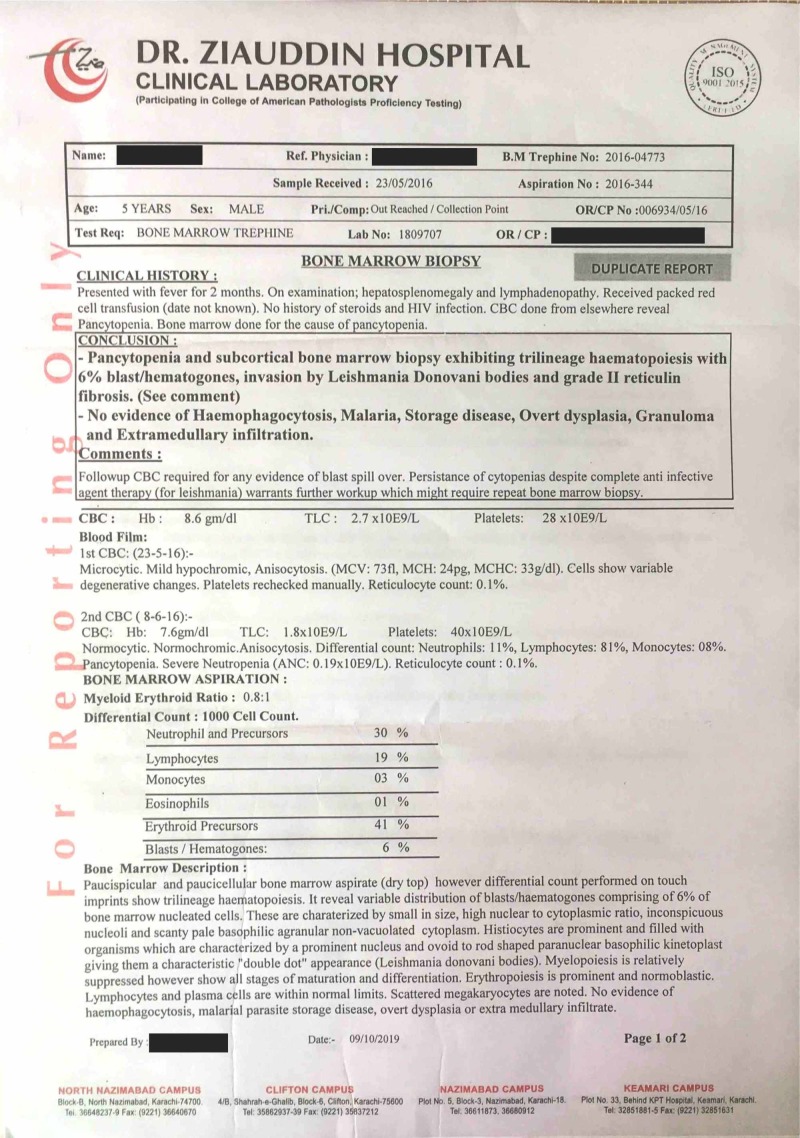
Bone marrow trephine biopsy report (before treatment) (page 1 of 2)

**Figure 2:**
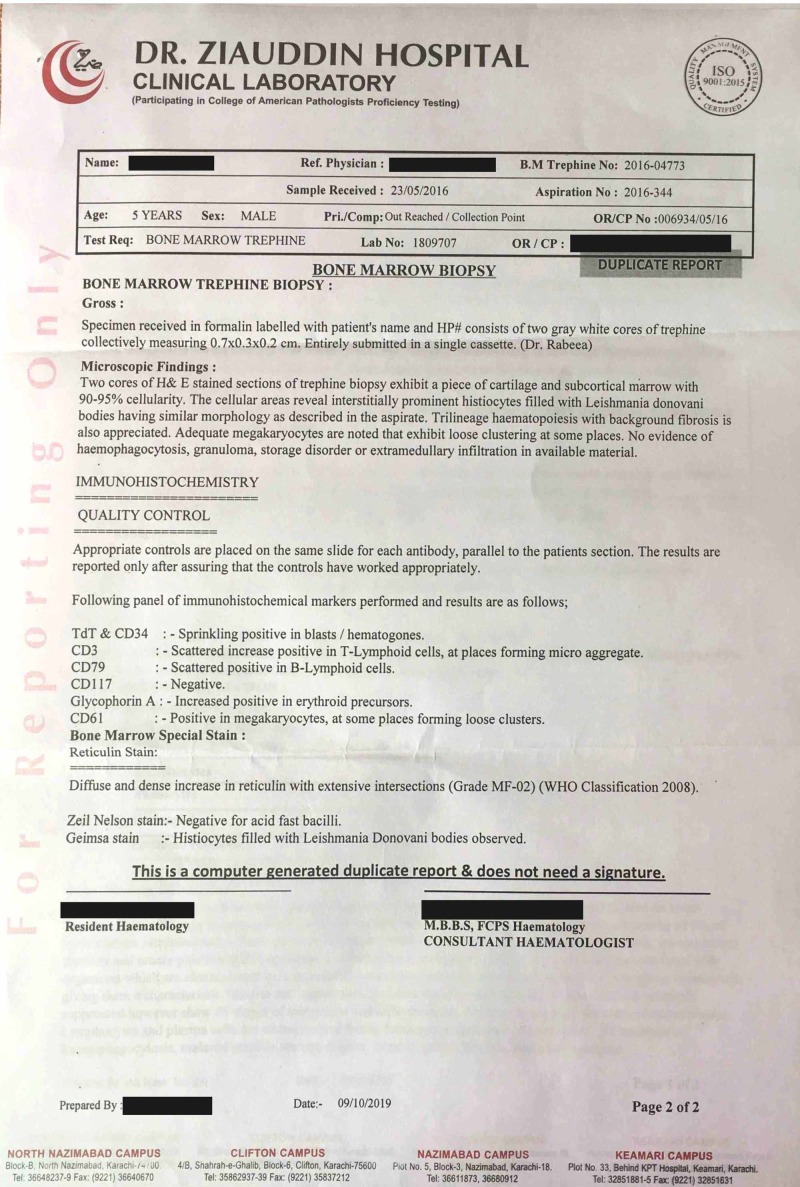
Bone marrow trephine biopsy report (before treatment) (page 2 of 2)

It is pertinent to mention the nonavailability of the actual pre-treatment biopsy images, as most laboratories in Pakistan do not provide biopsy images due to local laboratory policies and lack of specially adapted microscopes with a camera port that can take clear biopsy images due to a lack of funds and resources. The biopsy report is shown in Figures 1-2.

On presenting at Dr. Ruth KM Pfau Civil Hospital Karachi, the child had dark, pigmented, maculopapular rashes, which were fading, characteristic of measles, which he had developed a week ago. There was no history of vaccinations being carried out at any point in life. There was also no significant past medical history for blood transfusion or any chronic illnesses. The child was delivered by normal vaginal delivery at home and was a term baby. Labor was uneventful and the child cried immediately at birth. He was breastfed exclusively for two years and achieved all developmental milestones at an appropriate age. He was the fourth product of a consanguineous marriage. There was no history of tuberculosis in the family. There was no history of travel. The child belonged to a family with a poor socioeconomic status. They lived in a one-bedroom apartment, the sanitary conditions of which were poor and consumed unboiled water. His father worked as a driver in Swat.

On examination, the child looked pale with multiple hyperpigmented lesions present all over the body. There was marked conjunctival pallor with pedal edema extending up to the thighs and generalized lymphadenopathy. He was vitally stable. The chest was clear with normal vesicular breathing bilaterally and no cardiac murmurs could be appreciated on precordial exam. The child had a Glasgow Coma Scale (GCS) of 15/15 and was alert and well-oriented. On examination of the abdomen, the liver was palpable 5 cm below the right costal margin with a total span of 13 cm. The left lobe was palpable 7.5 cm below the xiphisternum. The spleen was palpable 9 cm below the left costal margin. Shifting dullness was also positive and gut sounds were audible.

Routine hematological investigations were carried out at presentation, which showed pancytopenia with a platelet count of 55000/ml and an absolute neutrophil count of 850. The peripheral film showed hypochromic, microcytic anisocytosis with pencil cells and macroovalocytes. Ultrasound reports confirmed hepatosplenomegaly. A bone marrow biopsy was not carried out again as the diagnosis had already been confirmed by the biopsy report from Ziauddin Hospital.

The patient was commenced on inj. amphotericin B deoxycholate intravenous (IV) 10 mg (1 mg/kg/day) under cover of inj. hydrocortisone 65 mg IV stat and inj. pheniramine maleate 13 mg IV stat. Over the course of the treatment, the child developed multiple episodes of bleeding per rectum for which he was transfused with packed red blood cells, platelets, and fresh frozen plasma at a dose of 10 ml/kg and inj. tranexamic acid 13 mg IV eight hourly. All doses were calculated based on the weight of the patient.

The regimen of inj. amphotericin B deoxycholate IV 10 mg, inj. hydrocortisone 65 mg IV, and inj. pheniramine maleate 13 mg was continued on an alternate-day basis for a further five weeks along with an oral daily regimen of syrup zinc sulfate 5 ml OD, syrup sucralfate 2.5 ml QDS, syrup calcium-P 5 ml TDS, and Pediasure milk sachet (as nutritional support). Steroid and anti-allergic medications were added, as the child developed a mild rash in response to the administration of amphotericin B. Questions from the Naranjo algorithm were asked to confirm if the development of the rash occurred due to the drug or some other contributing factor. However, the child did not have any history of allergies to foods or antibiotics and the rash disappeared once steroids and anti-allergic medications were added to the drug regimen.

The patient responded well to treatment after five weeks of therapy. The intended initial duration of treatment was three weeks. However, as symptoms (bleeding per rectum and generalized weakness) continued to persist, the treatment cycle was continued a further two weeks. His appetite improved significantly. Abdominal distention had decreased. There were no further episodes of bleeding per rectum or melena. Hemoglobin level increased from 6.1 mg/dl at the beginning of treatment to 9.0 mg/dl at the end of treatment. Platelet count improved to 269000/ml and absolute neutrophil count rose to 4171 cells per microliter.

The child was followed up on a monthly basis for six months. His general condition notably improved overtime and he regained weight in the ensuing weeks, after completion of treatment with nutritional buildup. Pedal edema also markedly improved at the first follow-up. Hepatosplenomegaly had reduced markedly as compared to the spans calculated at presentation.

## Discussion

There are three main forms of the condition leishmaniasis: visceral (VL, also known as kala-azar), cutaneous (CL), and mucocutaneous (MCL). Although the most prevalent form is the cutaneous one, the visceral form is difficult to detect, with a much longer incubation period and is fatal in 95% of cases if left untreated. An estimated 700000 to 1-million new cases and 20000 to 30000 deaths occur annually due to leishmaniasis overall, with approximately 50000 to 90000 new cases of VL occurring worldwide each year. In 2015, more than 90% of new cases reported to the World Health Organization (WHO) occurred in seven countries: Brazil, Ethiopia, India, Kenya, Somalia, South Sudan, and Sudan [5].

Cutaneous leishmaniasis and its related form, mucocutaneous leishmaniasis, collectively, though more common than the visceral form, have shorter incubation periods, have less morbidity and mortality, and are easier to diagnose as opposed to the visceral form. The latter form has an incubation period that can range from a few weeks to several months and may persist as an asymptomatic infection, showing its symptoms due to immunosuppression due to various causes and is usually fatal if left untreated [2].

In 2012, the WHO led an effort to report on the burden and distribution of the leishmaniases in 102 countries, areas, or territories worldwide. From the data available, the WHO estimated that 90% of global VL cases occurred in six countries: Bangla­desh, Brazil, Ethiopia, India, South Sudan, and Sudan. Of the global number of CL cases, >70% occurred in 10 countries: Afghanistan, Algeria, Brazil, Colombia, Costa Rica, Ethiopia, the Islamic Republic of Iran, Peru, Sudan, and the Syrian Arab Republic [6].

In 2015, data were collected on 25 countries declared leishmaniasis "high-burden" areas by the Global Leishmaniasis Programme, encompassing both cases of VL and CL, with the countries being dichotomized into their respective categories based on the dominant subtype of the condition seen. Under these statistical tabulations, Pakistan primarily came under a CL dominant region with only seven cases of VL being reported in 2014 and an incidence rate of 0.03 cases / 10000 inhabitants in endemic areas. It is also worth mentioning that according to WHO country profiles, the status for VL and CL in Pakistan is as an "endemic" condition [6-7]. According to WHO estimates for the year 2016, six countries, Afghanistan, Algeria, Brazil, Colombia, Pakistan, and the Syrian Arab Republic, each reported >10 000 CL cases, representing >70% of cases globally, further proving the dominance of CL in Pakistan [8].

Reported for the first time in Pakistan in 1960, VL is at high risk in the northern high-altitude (1500 meters and over) areas, namely Azad Jammu Kashmir (AJK), Gilgit-Baltistan Province, and Khyber Pakhtunkhwa (formerly Northwest Frontier Province), with most cases being children under six years of age with no known reservoir of infection. Currently, the exact incidence of VL in Pakistan is unknown, due to the absence of reporting programs. For CL, however, two main causative parasites have been identified: *L. major*, which mainly occurs in Balochistan and the neighbouring Punjab and Sindh provinces, and *L. tropica*, which has the widest distribution and is prevalent in urban areas of southern Punjab (Multan) and Balochistan (Quetta) but also focally in the northern areas. Multiple vectors, i.e., sandfly subtypes, have also been identified: *Phlebotomus​*​​​​​​ (*P.) hindustanicus, P. alexandri, P. longiductus, *and *P. kandelakii burneyi*. Considering the nature of VL incidence in Pakistan, the organism identified in one pediatric case of VL was *Leishmania infantosum *[9-10]. In a local study exploring the causative species of CL in Pakistan in 125 cases, *Leishmania tropica* was identified as the prevalent organism(89.2%) followed by* Leishmania major* (6.8%) and, unexpectedly, *Leishmania infantum *(4.1%) [11].

One of the major difficulties in the diagnosis of VL is its incubation period, ranging anywhere from 10 days to 34 months, but, on average, it's usually between three and eight months, with the main features being fever, weight loss, hepatosplenomegaly (usually spleen much larger than liver), skin hyperpigmentation, lymphadenopathy, pancytopenia, and hypergammaglobulinaemia [12].

The diagnosis of VL can be quite confusing and difficult due to a myriad of reasons; the differentials that it can commonly be mistaken for are malaria, typhoid, TB; co-infection with any of these conditions; the fact that the parasite localizes to the reticuloendothelial system; and the prolonged incubation period. A laboratory diagnosis of leishmaniasis can be made by the following: (i) demonstration of parasite in tissues of relevance by light microscopic examination of the stained specimen, in vitro culture, or animal inoculation; (ii) detection of parasite deoxyribonucleic acid (DNA) in tissue samples; or (iii) immunodiagnosis by the detection of parasite antigen in tissue, blood, or urine samples, by the detection of nonspecific or specific antileishmanial antibodies (immunoglobulin), or by assay for leishmania-specific cell-mediated immunity. A frequently employed method, which is also the "Gold Standard" for diagnosis, is to look for parasitic amastigotes in splenic or bone marrow aspirates, with sensitivities of 60%-85% and >95% respectively, along with their respective limitations. This method is rather convenient and relatively cheap in endemic regions of VL. However, there are various other methods, as mentioned earlier, but each of those involves specialized personnel, lab testing kits, and expensive equipment - all of which have their associated limitations in endemic areas [13].

Treatment for VL is dependent on various factors, such as host immune status, parasite subtype, drug sensitivity of the parasite, geographic location, and coexisting host conditions, as well as the host's health status overall [1]. Generally speaking, however, for the past seven decades, the mainstay of treatment for VL have been pentavalent antimonials as first-line medications and amphotericin B deoxycholate and pentamidine as the second line. In the previous decade, lipid forms of miltefosine, paromomycin, and amphotericin B have also been used. It is worth mentioning that the efficacy and dosage of all of the above medications have not been verified in all endemic areas. When treating patients for VL, it is vital to co-treat underlying conditions that may further improve prognosis: severe anemia with blood transfusions, co-infections with antimicrobials, nutritional supplementation, and adequate hydration. A patient is generally said to be cured if there is an absence of clinical relapse six months after treatment, with signs of treatment success being marked by the general well-being of the patient, regression of splenomegaly, improvement in blood parameters, and resolution of fever. Complete regression of splenomegaly, however, can take several months [14].

The pentavalent antimonials, sodium stibogluconate (SSG) and meglumine antimoniate are currently the first line in the treatment of VL in most parts of the globe, with >90% cure rates, except for Bihar in India and Nepal where resistance rates are up to 60%. They are chemically similar, and their toxicity and efficacy are related to their antimonial content: meglumine antimoniate solution contains 8.1% Sb5+ (81 mg/ml), whereas sodium stibogluconate solution contains 10% Sb5+ (100 mg/ml). Treatment with SSG involves injections at 20 mg/kg body weight, usually for 28-30 days (10) and with meglumine at a rate of 10-60mg/kg body weight for 12 days to three weeks (depending on the presence of a clinical/parasitological cure). Injections can be given IV in hospital settings and intramuscular (IM), albeit being very painful to administer, can be used if a large-scale treatment is being considered [14].

Miltefosine is an alkyl phospholipid (hexadecylphosphocholine) that was originally developed as an oral anticancer drug but was shown to have antileishmanial activity. When given at a dose of 2.5 mg/kg per day for four weeks in pediatric patients aged two to 11 years and for individuals 12 years and over at a dose of 50 mg/day for those weighing <25 kg, 100 mg/day for 25-50 kg body weight, and 150 mg/day for >50 kg body weight for 28 days, it has shown a cure rate of 94% in India and about 90% in Ethiopia [14].

Amphotericin B deoxycholate (a polyene antibiotic) and several of its related lipoidal formulations, including liposomal amphotericin B, amphotericin B lipid complex, and amphotericin B colloidal dispersion, have been used in treatment. They are similar to amphotericin B deoxycholate in their efficacy but are significantly less toxic [14]. When given daily or on alternate days by IV infusion in 5% dextrose for four hours at a dose of 0.75-1.0 mg/kg per day for 15-20 doses, amphotericin B deoxycholate was found to be 99% effective in India. It can be used in areas where the effectivity of SSG is <90% [15].

## Conclusions

Visceral leishmaniasis is one of the deadliest forms of leishmaniasis caused by a parasite of the genus Leishmania, which on improper, diagnosis can lead to mortality. The cutaneous form is more common in areas of the Khyber Pakhtunkhwa province of Pakistan. This may be the first reported case of successfully treated visceral leishmanias from that province. Diagnosis is confirmed by rapid diagnostic tests with bone marrow or splenic aspirates that demonstrate the characteristic Donovan bodies, and the disease can be successfully treated with intravenous sodium stibogluconate, amphotericin B, miltefosine, or antimony compounds. Our patient's symptoms improved with the administration of intravenous amphotericin B deoxycholate.
